# Oral drug suitability parameters†
†Electronic supplementary information (ESI) available: (1) A file containing the raw data for Table 1; (2) a file containing details of the mathematical equations associated to the PK models discussed; (3) a file containing the raw data for eqn (2); (4) a file containing the summary of raw data for Fig. 3 and 4a; (5) a file containing the summary of raw data for Fig. 4b; (6) a file containing the plot data for Fig. 4a and b, 5, 7 and 8a and b; (7) a file containing the command line code for the insilicolynxdqi Python library to replicate the raw data; (8) a file containing the required input data formatted for use with the insilicolynxdqi Python library. See DOI: 10.1039/c7md00586e


**DOI:** 10.1039/c7md00586e

**Published:** 2018-02-05

**Authors:** M. C. Wenlock

**Affiliations:** a InSilicoLynx Ltd , BioHub at Alderley Park , Mereside, Alderley Park , Cheshire , SK10 4TG , UK . Email: mark.wenlock@insilicolynx.com

## Abstract

Assessing the oral drug suitability of compounds as early as possible within drug discovery is an important objective. This study describes a methodology that attempts to simplify the evaluation of compounds based on their *in vivo* quantity levels within a mammalian body, represented using a mathematical model that imposes a time limitation on oral absorption and assumes non-instantaneous drug distribution between plasma and tissue. This simplification results in two new oral drug suitability parameters that can quantitatively relate oral dose to *in vivo* exposure for compounds with vastly different tendencies in terms of absorption into, and elimination from, the body. Consequently, the complexities associated with evaluating a compound's oral drug suitability are simplified to an assessment of these two new parameters. Application of this methodology at the virtual design stage is discussed, along with functionality that accounts for uncertainty related to a compound's distribution kinetics and errors associated to *in silico* QSAR predictions for the required input data.

## Abstract 2

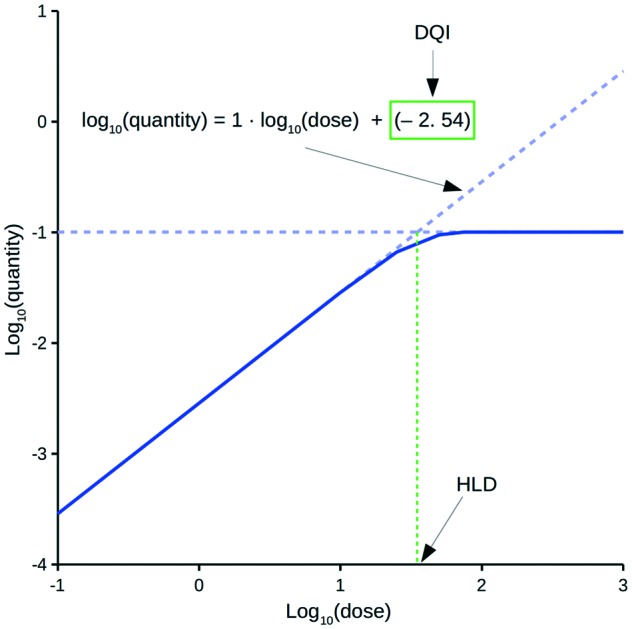
Assessing the oral drug suitability of compounds as early as possible is an important objective in drug discovery. Two new oral drug suitability parameters are proposed to facilitate the ranking of compounds with respect to dose and *in vivo* exposure.

## Introduction

When assessing the oral drug suitability of compounds it is common to consider physicochemical parameters such as hydrophobicity and molecular size.^1^,^2^ Over the past two decades, virtual drug design has been strongly influenced by hypotheses derived from the comparison of large sets of physicochemical property data associated to oral drugs;^3^–^9^ in particular, hypotheses that indicate limitations on a compound's predicted log *P*. However, such propositions tend to be ambiguous with regard to causality, as they rely heavily on arguments based on simple data trend observations. In practice, a compound's success as an oral drug in humans is predominantly defined by *in vivo* exposure, efficacy and toxicity factors; the evidence directly implicating a dependence of predicted log *P* on these *in vivo* factors is weak and influenced by the type of compounds being considered.^8^,^10^

The octanol–water partitioning system from which a log *P* is derived, and on which a predicted log *P* is based, is intended as a model system for compound transfer between aqueous and lipid phases and is arguably too simplistic for a whole mammalian system. This study addresses this point by proposing alternative oral drug suitability parameters for consideration during virtual drug design.

**Fig. 1:**
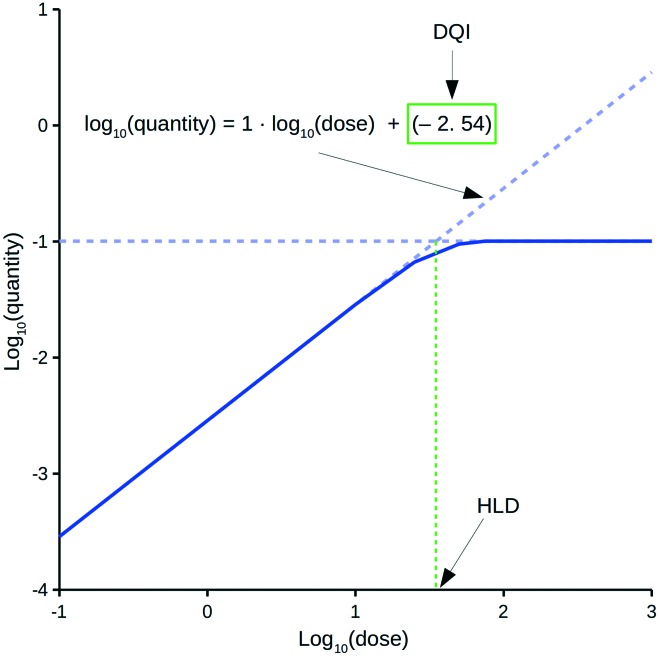
Example dose–quantity curve.

The oral drug suitability parameters are derived from rate equations for determining *in vivo* quantity levels associated to a relevant mathematical model of a mammalian system. The focus of this study is a mathematical model for a human system in the form of a three-compartment pharmacokinetic (PK) model, which imposes a time limitation on the absorption of an orally dosed drug from the small intestines, assumes non-instantaneous drug distribution between plasma and tissue, and only allows for elimination of the absorbed drug from one compartment.^11^,^12^ Application of the proposed PK model in a repeat-dosing simulation can approximate steady-state *in vivo* data for a quantity (*e.g.*, maximum plasma concentration) as a function of the oral dose, resulting in a dose–quantity curve (Fig. 1).

Each dose–quantity curve has two compound-specific features, which are treated as a pair of oral drug suitability parameters: the first is the dose–quantity intercept (DQI) for the regression line fitted through the curve's linear PK region; the second is the highest dose that still leads to linear PK (or near-linear PK) for the particular quantity (highest linear dose [HLD]). This study describes the methodology for calculating a compound's DQI and HLD, and demonstrates how to interpret the results to assess a compound's oral drug suitability. Importantly, the PK model is not intended to be physiologically accurate, rather it is a minimum model system that permits consideration of key factors related to a drug's *in vivo* exposure. Hence, the DQI and HLD contain information on the absorption, distribution and elimination properties of a compound based on this model system, which can facilitate the ranking of compounds. However, the calculated DQI and HLD values may differ from an *in vivo* measurement.

This study considers the application of the aforementioned PK model to 15 known oral drugs. The size of the set was restricted to 15 because of the limited number of compounds (within the literature and the European Bioinformatics Institute's ChEMBL database^13^) with experimentally derived values for p*K*_a_, aqueous solubility at pH 7.4 (and room temperature, solubility_pH7.4_), apparent human Caco2 membrane permeability (A to B) at pH 6.5 (*P*_app,Caco2,pH6.5_), human volume of distribution at steady state (*V*_ss_), human *in vivo* plasma clearance (Cl), human *in vivo* efficacy and, optionally, human plasma protein binding (PPB). However, it is envisaged that the DQI and HLD should be used at the virtual design stage and that such compound information would be generated using *in silico* QSAR models. Such methods have associated prediction errors, which can be large, so the impact of these errors on the DQI and HLD values is discussed.

## Experimental

### Compounds and required input data

Table 1 lists the 15 compounds being considered along with the following required input data: p*K*_a_, solubility_pH7.4_ (M), *P*_app,Caco2,pH6.5_ (cm s^–1^), *V*_ss_ (L kg^–1^), Cl (mL min^–1^ kg^–1^) and PPB (% bound). Further details can be found within the ESI.†

**Table 1:** Input data for 15 compounds (references within parentheses)

Drug	Charge type	p*K*_a_	Solubility_pH7.4_ (M)	*P*_app,Caco,pH6.5_ (cm s^–1^)	*V*_ss_ (L kg^–1^)	Cl (mL min^–1^ kg^–1^)	PPB (% bound)
Chlorpromazine	Monobase	9.24 (ref. 14 and 15)	3.72 × 10^–4^ (ref. 14, 15, 21 and 22)	2.00 × 10^–5^ (ref. 26)	10.00 (ref. 27)	16.00 (ref. 29)	98.64 (ref. 22)
Diazepam	Neutral		1.57 × 10^–4^ (ref. 22 and 23)	6.03 × 10^–5^ (ref. 26)	1.00 (ref. 27)	0.38 (ref. 29)	98.61 (ref. 22)
Diclofenac	Monoacid	4.26 (ref. 15–18)	6.53 × 10^–3^ (ref. 15, 16, 18, 23 and 24)	5.37 × 10^–5^ (ref. 26)	0.19 (ref. 27 and 28)	3.83 (ref. 28 and 29)	99.68 (ref. 22)
Furosemide	Monoacid	3.94 (ref. 14, 17 and 19)	1.78 × 10^–1^ (ref. 14)	2.82 × 10^–7^ (ref. 26)	0.11 (ref. 27 and 28)	2.19 (ref. 28 and 29)	98.37 (ref. 22)
Haloperidol	Monobase	8.63 (ref. 17 and 18)	7.56 × 10^–5^ (ref. 18, 21 and 22)	1.58 × 10^–5^ (ref. 26)	17.00 (ref. 27)	7.80 (ref. 29)	85.48 (ref. 22)
Imipramine	Monobase	9.50 (ref. 14 and 17)	9.85 × 10^–3^ (ref. 14)	2.75 × 10^–5^ (ref. 26)	12.00 (ref. 27)	13.00 (ref. 29)	87.87 (ref. 22)
Indomethacin	Monoacid	4.31 (ref. 15, 17 and 20)	1.44 × 10^–3^ (ref. 15, 20 and 25)	2.95 × 10^–5^ (ref. 26)	0.16 (ref. 27 and 28)	1.61 (ref. 28 and 29)	
Ketoprofen	Monoacid	4.25 (ref. 15–17 and 19)	5.55 × 10^–1^ (ref. 15 and 16)	4.68 × 10^–5^ (ref. 26)	0.13 (ref. 27)	1.60 (ref. 29)	98.73 (ref. 22)
Naproxen	Monoacid	4.23 (ref. 15–19)	9.87 × 10^–2^ (ref. 15, 16, 18 and 23)	4.68 × 10^–5^ (ref. 26)	0.09 (ref. 28)	0.07 (ref. 28)	
Nifedipine	Neutral		5.23 × 10^–5^ (ref. 18, 21, 22 and 24)	3.24 × 10^–5^ (ref. 26)	0.79 (ref. 27)	7.30 (ref. 29)	96.17 (ref. 22)
Phenytoin	Monoacid	8.06 (ref. 15–18)	8.51 × 10^–5^ (ref. 15, 16, 18, 21, 22 and 24)	3.98 × 10^–5^ (ref. 26)	1.40 (ref. 28)	0.57 (ref. 28)	87.11 (ref. 22)
Pindolol	Monobase	9.54 (ref. 15)	2.62 × 10^–2^ (ref. 15)	3.89 × 10^–5^ (ref. 26)	1.20 (ref. 27)	7.70 (ref. 29)	
Prazosin	Monobase	6.50 (ref. 17)	1.48 × 10^–5^ (ref. 22)	4.07 × 10^–6^ (ref. 26)	0.73 (ref. 27)	4.70 (ref. 29)	96.00 (ref. 22)
Trimethoprim	Monobase	7.26 (ref. 17)	2.17 × 10^–3^ (ref. 23)	8.71 × 10^–5^ (ref. 26)	1.50 (ref. 27)	2.10 (ref. 29)	66.10 (ref. 22)
Warfarin	Monoacid	4.97 (ref. 14, 15, 17 and 18)	3.35 × 10^–3^ (ref. 14, 15 and 18)	4.37 × 10^–5^ (ref. 26)	0.12 (ref. 27 and 28)	0.05 (ref. 28 and 29)	99.31 (ref. 22)

### PK model

**Fig. 2:**
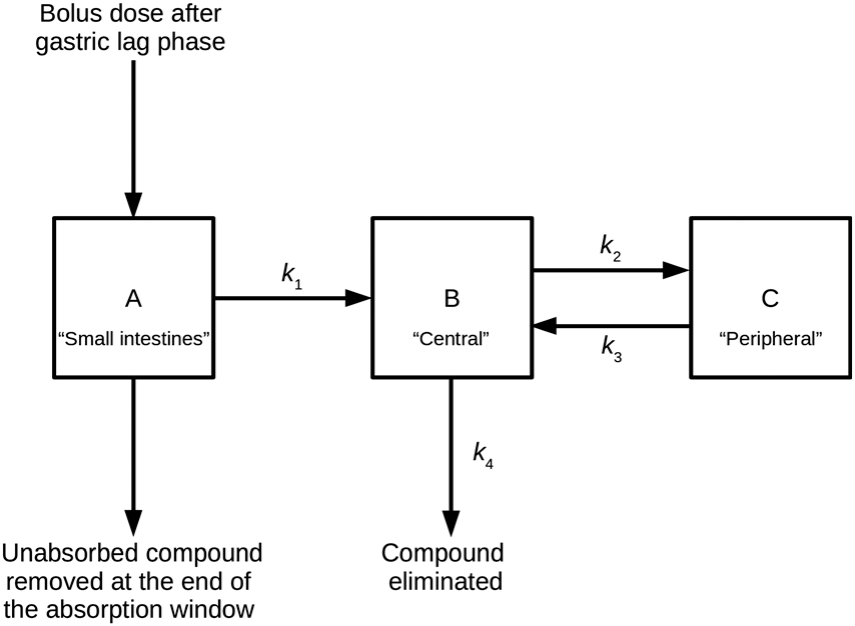
Representation of the compartmental PK model used.

The PK model combines a two-compartment model with an additional compartment to model absorption of extravascular doses of a compound from the small intestines.^11^,^12^ This model can be described by a series of linear differential equations that can be solved using the Laplace transformation technique to derive a series of rate equations.^30^Fig. 2 shows a compartmental representation of the model, and details of the mathematical equations can be found in the ESI.†

The application of the rate equations, along with the input data required to calculate compound compartment levels at different time points, constituted a simulation. Simulations were run at a series of doses and included multiple distribution kinetics scenarios. An open-source Python (software) library was written to perform and analyse these simulations.^31^

To establish steady-state conditions, simulations considered repeat dosing. Using the principle of superposition, compound compartment levels were considered for each repeated dose from time of dose to end of the simulation, and then all levels at each time point were summed for the repeated doses. Compartment A represented a hypothetical uniform cylindrical intestinal segment, filled with a constant intestinal fluid volume (*V*_intestinal_, L) of 0.08 L,^32^ into which the compound (in solid form) was delivered as a bolus; for this study, the intestinal fluid was represented by aqueous pH 6.5 buffer. The uniform mixing and dissolution process within compartment A was treated as instantaneous and only solubilised compound could undergo the one-way transfer into compartment B; this transfer was treated as either a zero-order or a first-order process, characterised by the absorption rate constant *k*_1_ (min^–1^), similar to the approach used by Dressman *et al.*^12^ The model restricted this process to an absorption window, during which compound could only be removed from compartment A by said absorption; any remaining compound at the end of the absorption window was discarded, and the model reverted to a classic two-compartment PK model.^33^ For clarity, the absorption window represented the transit of this uniform cylindrical slug of intestinal fluid (containing the dosed drug) through the small intestines, during which its shape and volume remained unchanged.

Compartment B (the central compartment) represented the body spaces into which a compound could distribute extremely rapidly (*i.e.*, plasma and well-perfused tissues, including the major eliminating organs). The volume of compartment B was compound specific and represented the compound's initial dilution volume (*V*_central_, L). Compartment C (the peripheral compartment) represented the body spaces into which a compound distributed more slowly. Compound transfer from compartment B to compartment C was treated as a first-order process, characterised by the rate constant *k*_2_ (min^–1^), and the reverse process was characterised by the rate constant *k*_3_ (min^–1^). It was assumed that, once absorbed, compound could only be removed permanently from the body from compartment B *via* a first-order process, characterised by the rate constant *k*_4_ (min^–1^).

### Simulation time frame

Each simulation spanned 168 h and comprised 14 repeat doses of similar size for each compound. Each simulation time frame consisted of a start time (*t*_0_, min) and an end time (*t*_end_, min). With respect to the *i*th dose, *t*_*i*_ (min) was the time that this dose was given, and the first dose (*t*_1_) was equal to *t*_0_. The time of subsequent doses (*t*_2_, *t*_3_, *etc.*) was 12 h after the previous dose and reflects the dosing interval. Mathematically, each dose was considered in isolation, where the amounts of compound in the PK model's three compartments was zero at *t*_*i*_. A bolus of the *i*th dose appeared in compartment A at time *t*_*i*_,_A_, where the time difference between *t*_*i*_ and *t*_*i*,A_ represented an absorption delay of 1 h to reflect the gastric lag phase.^34^ The absorption window started at *t*_*i*,A_ and ended at time *t*_*i*,B_, and equated to 4 h.^35^,^36^ Compound in compartment B was transferred to compartment C or removed from further consideration during the time between *t*_*i*,A_ and *t*_end_. This time period also reflected the time during which compound in compartment C could be transferred to compartment B.

### Saturation state of compartment A

For scenarios where *V*_intestinal_ was saturated with compound at *t*_*i*,A_, a constant rate of compound transferred from compartment A to compartment B applied, given by *k*_1_ multiplied by the saturated amount of compound in the intestinal fluid (mg). The latter was derived from the compound's aqueous solubility at pH 6.5 (solubility_pH6.5_, M) multiplied by its molecular weight and *V*_Intestinal_. This rate of transfer was considered up to time *t*_*i*,B_ or *t*_*i*,A,unsaturated_, when *V*_intestinal_ became unsaturated. At *t*_*i*,A,unsaturated_ the amount of compound remaining in compartment A reflected its solubility_pH6.5_, from which point the rate of transfer followed first-order kinetics until time *t*_*i*,B_.

The solubility_pH6.5_ was calculated by multiplying a compound's solubility_pH7.4_ by the ratio of its fraction neutral at pH 7.4 to its fraction neutral at pH 6.5; the fraction neutral values were calculated using the method described by Wenlock.^37^

### Simulation time points

Each simulation was defined by approximately 1000 time points. This included time points at 15 min intervals between *t*_0_ and *t*_end_, along with 336 randomly selected time points from the same time frame. The following specific time points were also considered: *t*_0_, *t*_*i*_, *t*_*i*,A_, *t*_*i*,B_, *t*_end_ and, where applicable, *t*_*i*,A,unsaturated_.

### Estimating *k*_1_

Estimations were based on a compound's predicted human jejunal effective permeability at pH 6.5 (*P*_eff,human,pH6.5_, cm s^–1^) from a uniform cylindrical intestinal segment, filled with intestinal fluid equal to *V*_intestinal_, using the following equation:

1 *k*_1_ = (SA·*P*_eff,human,pH6.5_·60)/*V*_intestinal_

where SA is the surface area of the cylindrical intestinal segment.^38^ For a uniform cylinder, *V*_intestinal_ is equal to π*r*^2^*l* and SA equals 2π*rl*, where *r* is the radius equal to 1.25 cm and *l* is the length.^35^,^39^ To account for intestinal folds, the value of SA was further multiplied by an absorption amplification factor of 2.^39^,^40^

*P*_eff,human,pH6.5_ was estimated based on a compound's *P*_app,Caco2,pH6.5_ using a similar approach to that previously described.^37^,^41^ Critically, this approach focuses on the establishment of a (log_10_–log_10_) linear regression equation for a set of compounds between their neutral species human membrane permeability at pH 6.5 (*P*_m,human,neutral,pH6.5_, cm s^–1^) and their neutral species human Caco2 membrane permeability (A to B) at pH 6.5 (*P*_m,Caco2,neutral,pH6.5_, cm s^–1^). For this study, a larger set of 32 compounds was used to establish the following regression equation:

2 log_10_(*P*_m,human,neutral,pH6.5_) = 0.916·log_10_(*P*_m,Caco2,neutral,pH6.5_) + 1.579

where *r*^2^ = 0.88. The data for this regression equation were predominantly sourced from Avdeef and Tam,^19^ and supplemented with data from Sjögren *et al.*^41^ The charge type at pH 6.5 of the compounds considered was either monoacidic, monobasic, neutral or zwitterionic. Further details can be found within the ESI.†

### Fraction escaping first-pass metabolism (*F*_h_)

The model assumes that drug absorption is exclusively through the small intestines, with no gut wall metabolism, and that *F*_h_ can be estimated using the equation:^42^

3 *F*_h_ = 1 – Cl_b_/*Q*_h_

where *Q*_h_ (mL min^–1^) is the rate of liver blood flow and Cl_b_ is the human *in vivo* blood clearance (mL min^–1^ kg^–1^) for a compound. This study used a value of 1450 mL min^–1^ for *Q*_h_ in humans and a body weight of 70 kg. The model assumes that the ratio of blood concentration to plasma concentration equals 1 for all of the compounds considered. Hence, Cl is assumed to equal Cl_b_.

### Distribution kinetics scenarios and estimates for *k*_2_, *k*_3_ and *k*_4_

Three volume of distribution terms are relevant for each compound considered: *V*_central_, *V*_ss_ and the terminal volume of distribution (*V*_terminal_, L). A compound's *V*_ss_ value is one of the model's required input data values (see Table 1). With respect to *V*_central_ and *V*_terminal_, each simulation considers five distribution scenarios: (i) *V*_central_ equals 3.0 L, and the ratio of *V*_terminal_ to *V*_ss_ equals 1.1; (ii) *V*_central_ equals 50% of the *V*_ss_, and the ratio of *V*_terminal_ to *V*_ss_ equals 1.1; (iii) *V*_central_ equals 3.0 L, and the ratio of *V*_terminal_ to *V*_ss_ equals 2.0; (iv) *V*_central_ equals 50% of the *V*_ss_, and the ratio of *V*_terminal_ to *V*_ss_ equals 2.0; and (v) *V*_central_ equals the mid-point between the two previous values of *V*_central_, and *V*_terminal_ equals the mid-point between the two previous values of *V*_terminal_. The first four scenarios represent possible extreme values and the fifth represents a mid-point value for a compound.

For each scenarios, values for *k*_2_, *k*_3_ and *k*_4_ were derived using the values for *V*_central_, *V*_ss_, *V*_terminal_ and Cl, in conjunction with rearrangement of standard mathematical equations associated to a two-compartment model.^33^ Details of these equations can be found in the ESI.†

Five simulations were performed for each compound and each dose, based on the five scenarios.

### Doses considered

Simulations were run at 20 different oral doses (mg): 0.000001, 0.000005, 0.00001, 0.00005, 0.0001, 0.001, 0.01, 0.1, 1, 10, 25, 50, 75, 100, 250, 500, 1000, 2500, 5000, 10 000.

### Quantities calculated

A simulation resulted in estimates of quantities of drug in compartments B and C at different time points. For each dose, the compound levels at steady state in compartment B were calculated, including the maximum concentration (*C*_ss,central,max_, mg L^–1^) and area under the curve (AUC_ss,central_, mg min L^–1^). Steady-state conditions were assumed to have been reached by the time of the last repeat dose in a simulation. The AUC_ss,central_ was calculated using the composite Simpson's rule, integrating over the time course of the last repeat dose. Free levels were calculated by multiplying the total *C*_ss,central,max_ or AUC_ss,central_ by the fraction unbound (*i.e.*, (100 – % bound)/100).

The quantities calculated for each simulation were collated and used to create a (log_10_–log_10_) dose–quantity curve for each distribution kinetics scenario.

### Estimating DQI and HLD

For a set of dose–quantity data, the DQI equals the intercept for a linear regression equation through the region of the log_10_(dose) *versus* the log_10_(quantity) curve, where the slope was approximately equal to 1 (within ±0.0001). The HLD was the log_10_(dose) calculated using said linear regression equation and the maximum value of the log_10_(quantity) data (*i.e.*, log_10_(max_quantity)). Typically, this maximum value reflected the level of the horizontal plateau (Fig. 1). In the few cases where no plateau was observed, the maximum value was that of the highest dose considered.

### Assessing the impact of errors on DQI and HLD

Variations in DQI and HLD values associated to total level quantities, based on errors in *V*_ss_ and Cl values for each compound, were also considered. An additional 50 scenarios were calculated for each compound, where original values for these two parameters were replaced by values randomly selected from a Gaussian distribution. Each distribution was based on the log_10_(original value) and a standard deviation value of 0.3.

### Presentation of DQI and HLD data

Each set of DQI and HLD data was treated as a Cartesian coordinate pair; due to the five distribution kinetics scenarios, each compound had five pairs. Where errors were considered, each compound had 255 coordinate pairs (*i.e.*, five distribution kinetics scenarios for each of the 51 input data scenarios). Non-self-intersecting closed polygons, whose vertices were described using these Cartesian coordinates in the DQI–HLD plane, were used to represent each compound. The centroid and area of each polygon were calculated using standard methods. Again using standard methods, the second moments of area of each polygon were calculated to reflect the distribution of vertices in the *x*-axis (*i.e.*, HLD dimension) and *y*-axis (*i.e.*, DQI dimension).

## Results and discussion

### DQI and HLD parameters

The proposed oral drug suitability parameters are intended to provide insight into the *in vivo* quantities of a compound based on a relevant PK model of a mammalian system. For the purpose of this study, steady-state conditions are of interest, and 14 repeat doses within a simulation are considered sufficient to establish such. Importantly, the DQI and HLD parameters are composite terms that simplify the understanding of how a compound with a given p*K*_a_, solubility_pH7.4_, *P*_app,Caco2,pH6.5_, *V*_ss_, Cl and (in the case of free levels) PPB profile behaves within the PK model, facilitating dose predictions.

Fig. 1 highlights a typical dose–quantity curve resulting from the application of the PK model. It is characterised by a linear region with a slope of 1 for doses below a certain limit. As the dose increases above this limit, compartment A becomes increasingly saturated for the duration of the absorption window. This results in the dose–quantity curve bending as the dose increases, eventually plateauing. For the linear region, the corresponding regression equation takes the form of:

4 log_10_(quantity) = 1·log_10_(dose) + DQI

Rearrangement of eqn (4) leads to eqn (5) or (6):

5 quantity = dose·10^DQI^

6
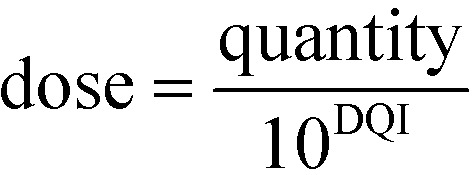



However, these equations only apply up to a log_10_(dose) equal to the HLD; this equates to the extrapolated log_10_(dose), using eqn (4), for the log_10_(max_quantity) observed for a particular distribution kinetics scenario (Fig. 1). The HLD value arrived at using this method is not strictly the HLD, as the dose–quantity curve has already begun to deviate from linearity at this log_10_(dose) (Fig. 1); rather, it is an approximation using a standardised approach.

Importantly, the DQI and HLD provide a simple way to understand a compound's dose–quantity curve. A plot of DQI against HLD provides an alternative scale against which to evaluate different compounds.

### PK model

The PK model used in this study is considered the minimum necessary to account for time-limited oral absorption, non-instantaneous drug distribution between plasma and tissue, and elimination from the central compartment (B). The PK model is intended to be a generic model for use at the virtual drug design stage, and the model settings described in the Experimental section are intended to have broad applicability. Importantly, the PK model and the associated constraints may not be optimal for all compounds. The approach used for modelling oral absorption is based on that used by Dressman *et al.*^12^ It is simplistic in nature but deemed sufficient for use at the virtual drug design stage. More complex models are available and Sjögren *et al.* can provide further insight.^41^ For clarity, the absorption window is necessary to account for non-linear PK resulting from compound absorption limitations, and the calculation of a compound's HLD is dependent on this feature.

Other PK models based on this linear differential equation approach are possible – rate equations for some alternative PK models can be found in the ESI.† The simplest of these is a one-compartment intravenous (bolus dosing) model that only considers a first-order rate constant for elimination.^43^ This model can be extended to include an oral absorption step – the approached used by Wenlock and Page.^37^,^42^ Such a model is applicable for compounds where oral absorption cannot be assumed to be instantaneous; it can also be refined to account for time-limited oral absorption, and details of this can be found in the ESI.† A limitation of one-compartment models is the assumption of instantaneous drug distribution between plasma and tissue; this is overcome in the present PK model by use of a second compartment that permits distribution kinetics to be modelled. For reference, details of a two-compartment intravenous model can be found in the ESI.†

### Distribution kinetics scenarios

Treating distribution of absorbed drug as not being instantaneous can lead to significant variations in calculated *in vivo* quantities.^33^ Specifically, the model assumes non-instantaneous distribution between compartments B and C, albeit distribution within each compartment is instantaneous.

**Fig. 3:**
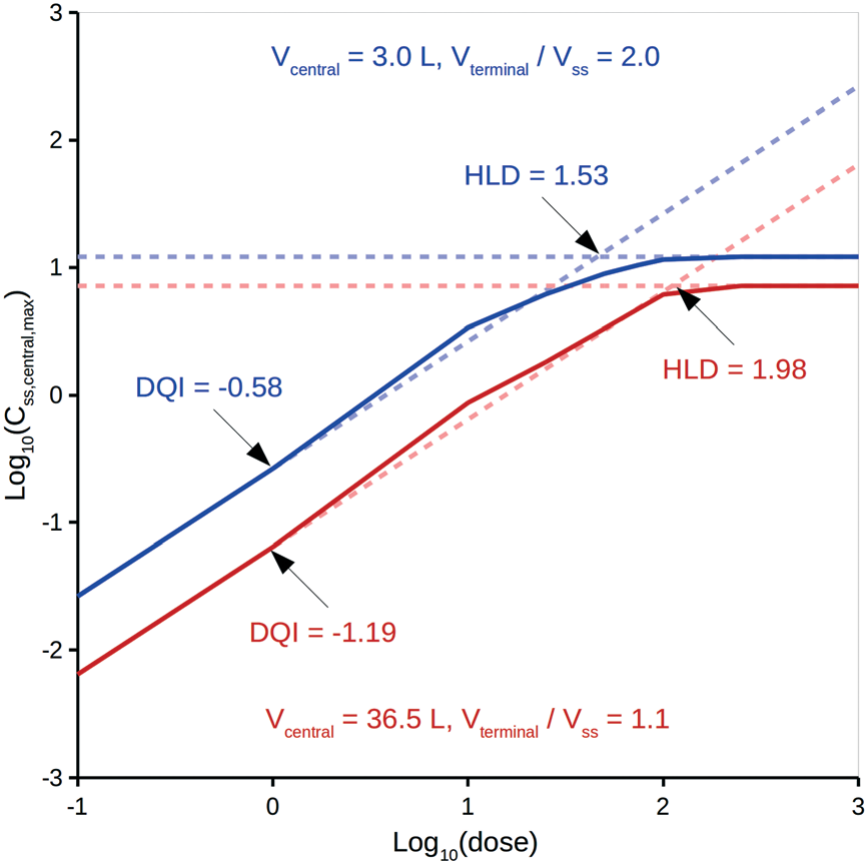
Log_10_(dose) *versus* log_10_(*C*_ss,central,max_) for two distribution kinetics scenarios for diazepam: blue curve, *V*_central_ = 3.0 L, *V*_terminal_/*V*_ss_ = 2.0; red curve, *V*_central_ = 36.5 L, *V*_terminal_/*V*_ss_ = 1.1.

It is intended that DQI and HLD be calculated at the virtual drug design stage using *in silico* technologies^37^,^44^ to provide the input data, but, for simplicity, this study only considers experimentally derived input data. With respect to a compound's distribution kinetics, it is unlikely that there will be any insight into these at the virtual design stage. To account for this, the model considers five hypothetical distribution kinetics scenarios for each compound, intended to cover a range of possibilities that encompass the true situation. These scenarios depend on a range of hypothetical values for a compound's *V*_central_ and *V*_terminal_. *V*_central_ ranges from 3.0 L, which is the approximate value for the plasma volume in human, to a value that is 50% of the compound's *V*_ss_; a mid-point value is also considered. *V*_terminal_ ranges from 1.1 times the *V*_ss_ to 2.0 times the *V*_ss_; a mid-point value is also considered. For comparison, Rowland and Tozer^33^ provide human details for aspirin, salicylic acid and gentamicin C: their *V*_central_ values can be calculated as 5.3, 6.8 and 14.0 L, respectively, and their *V*_terminal_ values as 1.1-, 1.3- and 5.1-fold greater than *V*_ss_, respectively. The *V*_terminal_ of gentamicin C is considered an extreme and a value 2.0-fold greater than *V*_ss_ deemed appropriate. To illustrate the effect of these different distribution kinetics scenarios, Fig. 3 shows the dose *versus C*_ss,central,max_ curves generated by the PK model for two of these scenarios for diazepam (further details can be found in the ESI†).

It can be seen from Fig. 3 that differing distribution kinetics scenarios can lead to significant variation in values for DQI (*i.e.*, 2.1-fold) and HLD (*i.e.*, 1.3-fold).

### Visualising DQI and HLD data

**Fig. 4:**
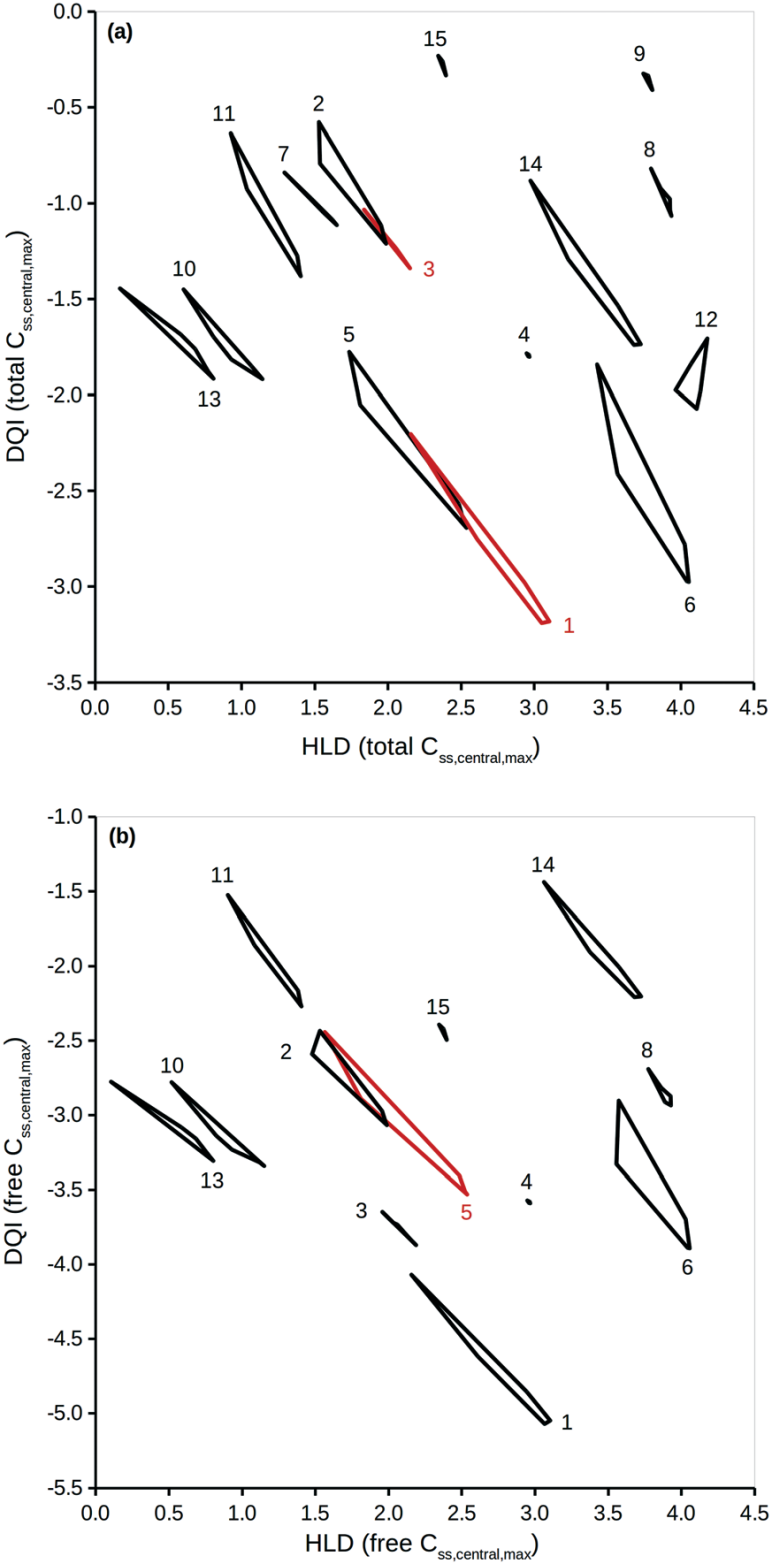
DQI *versus* HLD for *C*_ss,central,max_ (a) total levels and (b) free levels. Each polygon is made up of DQI and HLD data associated to the simulations for five different distribution kinetics scenarios. The red polygons have no meaning – the colour is used to aid visualisation of the different polygons. The numbers refer to the following compounds: 1, chlorpromazine; 2, diazepam; 3, diclofenac; 4, furosemide; 5, haloperidol; 6, imipramine; 7, indomethacin; 8, ketoprofen; 9, naproxen; 10, nifedipine; 11, phenytoin; 12, pindolol; 13, prazosin; 14, trimethoprim; 15, warfarin.

To properly reflect the lack of knowledge regarding a compound's distribution kinetics at the virtual design stage it is important to consider the DQI and HLD from each of the five distribution kinetics scenarios. A way of visualising this information is to treat each pair of DQI and HLD values as a Cartesian coordinate within the DQI–HLD plane, to represent a vertex of a non-self-intersecting closed polygon. Fig. 4 shows such a plot for the *C*_ss,central,max_ total levels for the 15 compounds considered, and a similar plot for the *C*_ss,central,max_ free levels (further details can be found in the ESI†).

The polygons shown in Fig. 4 highlight the DQI–HLD space occupied by a compound. The extent of the area overlap of the polygons for two different compounds reflects their similarity. Importantly, at the virtual design stage it is not possible to define precise values for a compound's DQI and HLD. Instead, it is possible to determine an area of DQI–HLD space, defined by a non-self-intersecting closed polygon, where the true value lies. Provided that the two polygons do not overlap, the compounds can be argued to have distinct DQI and HLD values.

**Fig. 5:**
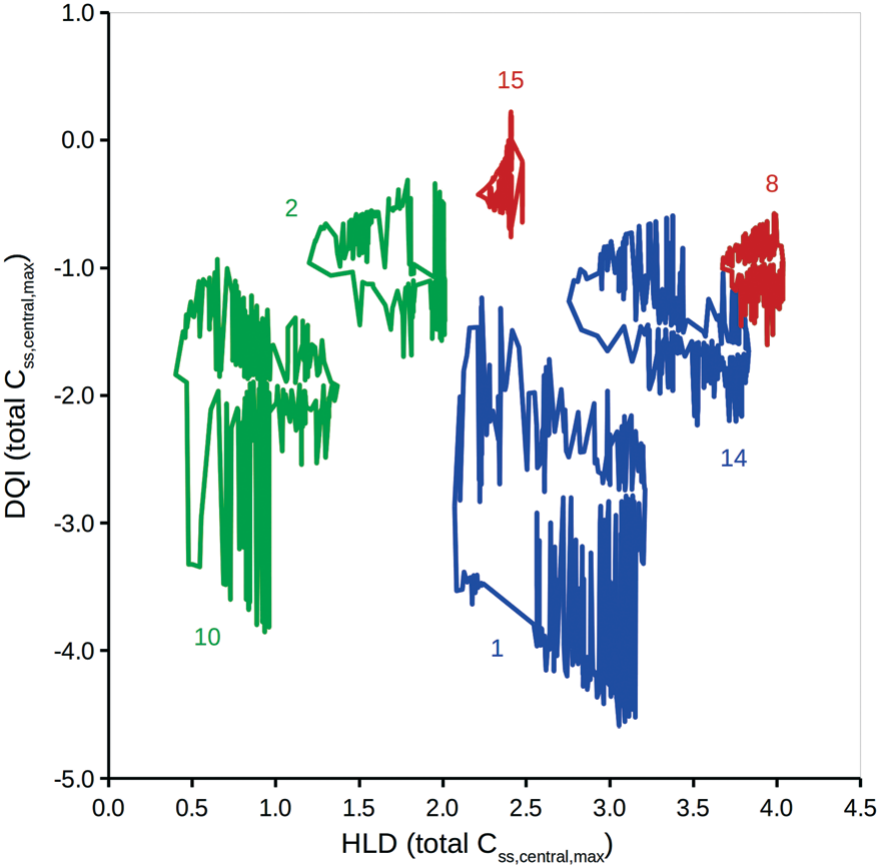
DQI *versus* HLD for total levels of *C*_ss,central,max_. Each polygon consists of DQI and HLD data associated to the simulations for five different distribution kinetics scenarios using 51 input data error scenarios. The colours represent charge types: green, neutral; red, monoacid; blue, monobasic. The numbers refer to the following compounds: 1, chlorpromazine; 2, diazepam; 8, ketoprofen; 10, nifedipine; 14, trimethoprim; 15, warfarin.

This method can be expanded to incorporate errors in the estimation of the input data. A Gaussian distribution can be assumed, based on the original value and an estimation of the associated standard deviation. Additional simulations using randomly selected values from such a Gaussian distribution can then be considered. To illustrate, DQI and HLD were calculated for the 15 compounds, using 50 additional simulations to account for an error of 0.3 on a logarithmic base 10 scale for the values of *V*_ss_ and Cl. To simplify the plot in Fig. 5, DQI–HLD polygons are shown for only six compounds: chlorpromazine (monobase), diazepam (neutral), ketoprofen (monoacid), nifedipine (neutral), trimethoprim (monobase) and warfarin (monoacid) (further details can be found in the ESI†). It is clear from Fig. 5 that the polygons are very irregular, but again, provided that two polygons do not overlap, the compounds can be argued to have distinct DQI and HLD values. This approach also applies when the input data are sourced from *in silico* QSAR models, where any prediction error is expressed as a standard deviation.^45^

The areas covered by the different polygons vary, with that for chlorpromazine being the largest and that for warfarin the smallest. The calculated areas are summarised in Table 2, along with the polygons' second moment of area and centroid values in the DQI and HLD dimensions. The second moment of area indicates the sensitivity of the compound to errors in the input data with respect to the DQI and HLD dimensions; the larger the value, the greater the sensitivity. With respect to warfarin, the DQI value is more sensitive to the combined errors in the values of *V*_ss_ and Cl than that for HLD; the reverse is true for prazosin. It is useful to think of the HLD dimension reflecting the ability of a compound to be absorbed into the body, while the DQI dimension reflects the extent of elimination of a compound from the body.

**Table 2:** Summary of the total level *C*_ss,central,max_ DQI–HLD polygon details associated to the simulations for five different distribution kinetics scenarios using 51 input data error scenarios

Drug	Area	Second moment of area		Centroid	
		HLD	DQI	HLD	DQI
Chlorpromazine	1.72	15.30	11.88	2.61	–2.93
Diazepam	0.33	0.31	0.89	1.63	–0.94
Diclofenac	0.73	1.82	3.04	2.03	–1.56
Furosemide	0.09	0.36	0.74	2.93	–2.03
Haloperidol	1.58	13.27	5.88	1.90	–2.83
Imipramine	1.00	9.68	13.96	3.74	–3.06
Indomethacin	0.20	0.21	0.47	1.52	–1.02
Ketoprofen	0.11	0.11	1.63	3.88	–1.00
Naproxen	0.05	0.01	0.70	3.72	–0.45
Nifedipine	0.78	3.54	0.51	0.77	–2.07
Phenytoin	0.46	0.62	0.60	1.11	–1.13
Pindolol	0.33	1.32	5.18	3.98	–1.95
Prazosin	0.80	4.35	0.17	0.39	–2.30
Trimethoprim	0.49	0.99	5.28	3.26	–1.39
Warfarin	0.05	0.01	0.29	2.38	–0.30

**Fig. 6:**
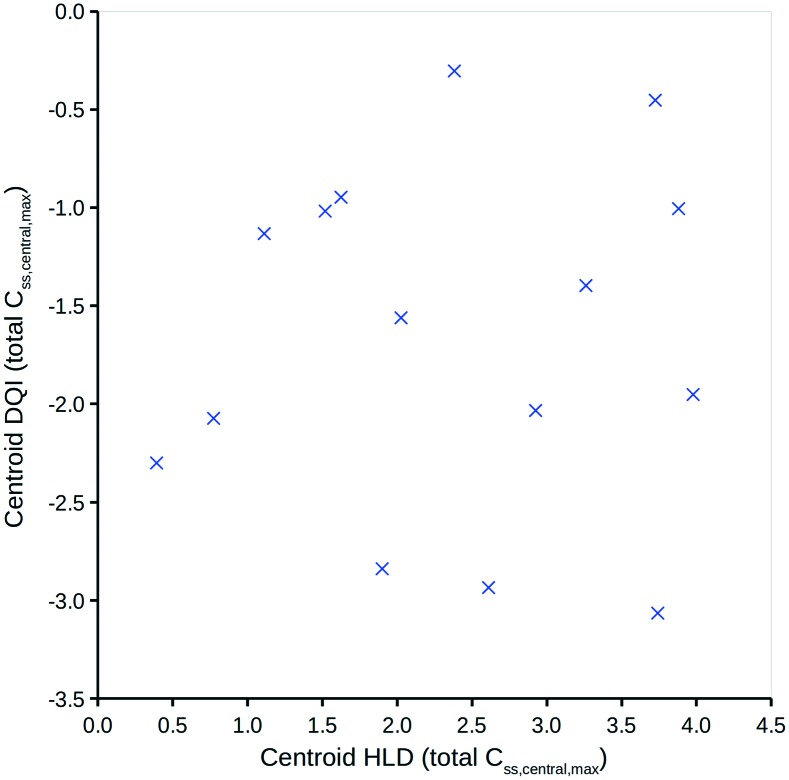
Total level *C*_ss,central,max_ centroid DQI *versus* centroid HLD.

To simplify the information represented by the polygons in Fig. 5, it is proposed that the centroid coordinate could be used to standardise comparisons. Fig. 6 shows a DQI–HLD plot for the total level *C*_ss,central,max_ centroid values for the 15 compounds considered.

### Interpreting the DQI and HLD oral drug suitability parameters

Fig. 6 shows how different compounds have vastly different tendencies in terms of absorption and elimination. Absorption is indicated by a compound's centroid value on the HLD dimension, and elimination by a compound's centroid value on the DQI dimension. Arguably, compounds with a higher DQI (*i.e.*, lower elimination) are preferable, but a higher HLD can compensate for a lower DQI. Consider the two monobases prazosin and chlorpromazine: prazosin has a higher centroid DQI (total *C*_ss,central,max_) of –2.30, compared with –2.93 for chlorpromazine, but a lower centroid HLD (total *C*_ss,central,max_) of 0.39 compared with 2.61 for chlorpromazine. If the HLD value is used as the log_10_(dose) in eqn (4), as shown in eqn (7) (where the slope of 1 is omitted):

7 log_10_(max_quantity) = HLD + DQI

then the log_10_(total *C*_ss,central,max_) for prazosin is –1.91 compared with –0.32 for chlorpromazine. It follows that better absorption can compensate for higher elimination.

These observations are well established, but evaluation of the properties of oral compounds (*i.e.*, p*K*_a_, solubility_pH7.4_, *P*_app,Caco2,pH6.5_, *V*_ss_, Cl and, optionally, PPB) can be vastly simplified to a quantitative comparison within the two dimensions of DQI and HLD. The magnitude of a compound's DQI and HDI should be considered in conjunction with the desired log_10_(quantity).

### Proposed use of DQI and HLD within virtual drug design

It is envisaged that input data for the present PK model would be sourced from *in silico* QSAR models.^37^,^44^ At the virtual design stage many potential compounds can be considered and it is proposed that their DQI–HLD polygon areas be calculated as described (including consideration of errors in the estimations of the input data).^45^ The results of these calculations can be visualised as described to give insight into the polygon DQI–HLD space occupied by different compounds. These compounds can also be ranked by application of eqn (5) or (7). For a target log_10_(max_quantity) requirement, eqn (7) can be used to estimate the log_10_(max_quantity) for each compound; if this exceeds what is needed, a lower log_10_(dose) will suffice. However, if the calculated log_10_(max_quantity) is less than what is needed, then such compounds are not viable as absorption limitations would prevent the use of a higher dose. Similarly, if compounds need to be ranked on the magnitude of a quantity at a particular dose, *e.g.*, 50 mg, eqn (5) could be applied using a value of 50 mg for compounds with an HLD ≥1.70 (*i.e.*, log_10_(50)) and an anti-log_10_(HLD) value for those compounds with an HLD <1.70.

### DQI and *in vivo* efficacy

The DQI is a single factor that relates dose to a compound's *in vivo* (steady-state) quantity following repeat oral dosing in a model system representation of the body. It can be considered a measure of a compound's *in vivo* exposure.

**Fig. 7:**
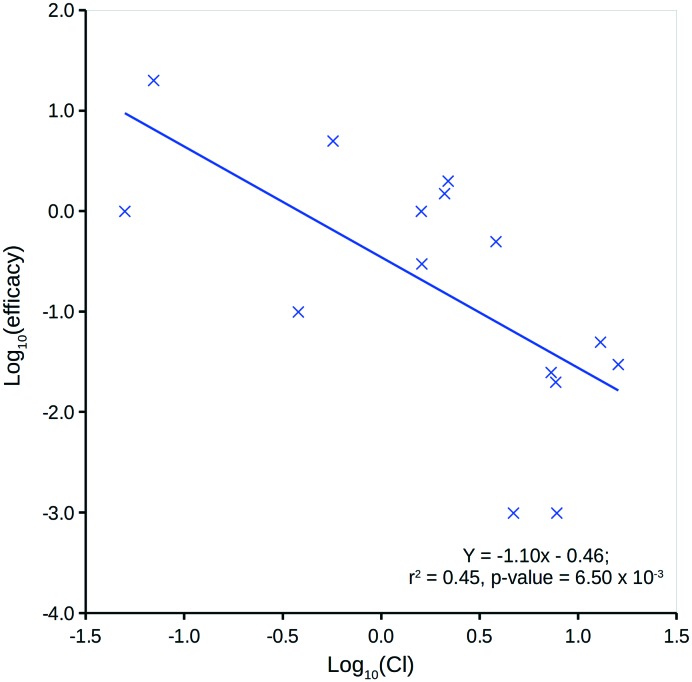
Log_10_(Cl) *versus* human *in vivo* log_10_(efficacy).

Human *in vivo* efficacy (mg L^–1^) data for the 15 compounds considered have been sourced from Schulz *et al.* (further details can be found in the ESI†).^46^ Comparison to predicted log *P* (ref. 13) indicates a very weak, non-significant linear relationship, but comparison to Cl shows a significant linear relationship (Fig. 7). This is not unexpected, as a compound's Cl will heavily influence *in vivo* levels – with respect to an intravenous one-compartment model, the steady-state concentration upon repeat dosing is, in theory, inversely proportional to Cl.^43^

**Fig. 8:**
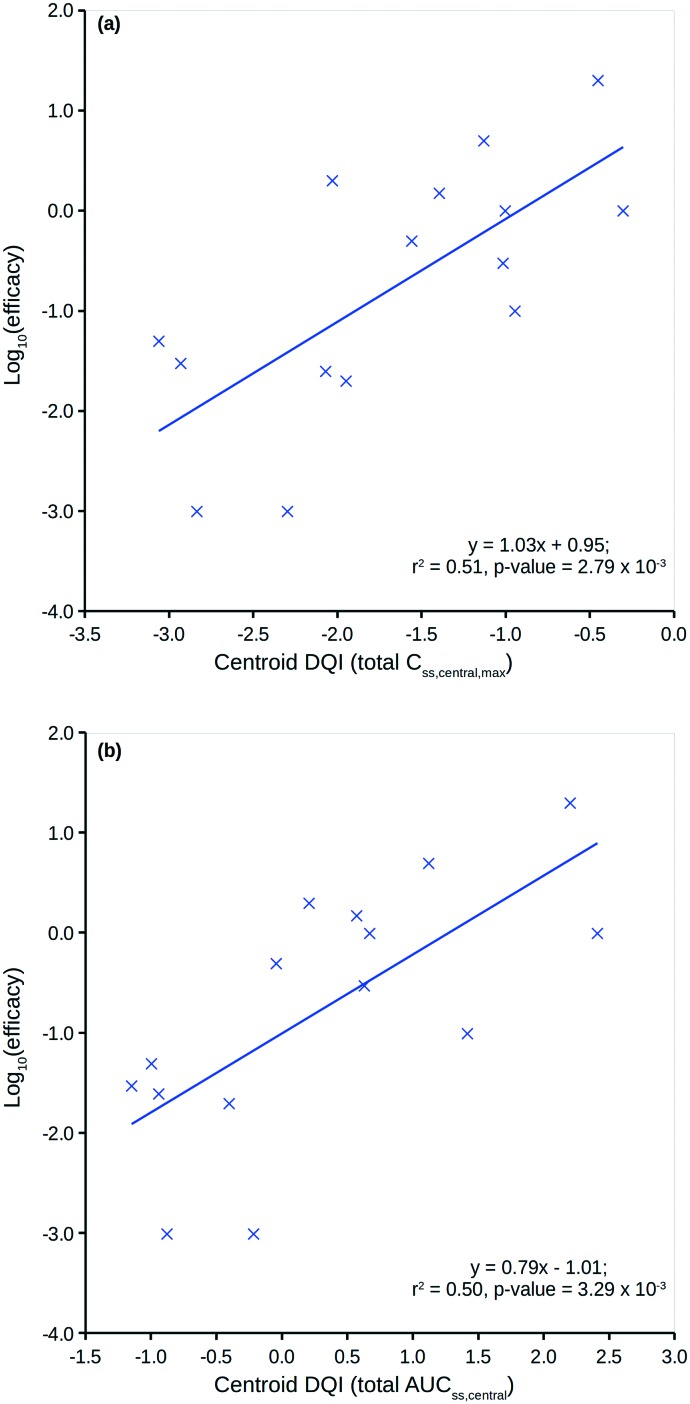
Human *in vivo* log_10_(efficacy) *versus* the total level centroid DQI for (a) *C*_ss,central,max_ and (b) AUC_ss,central_.

Fig. 8 shows two plots between the 15 compounds' human *in vivo* efficacy and their centroid DQI for (a) total level *C*_ss,central,max_ and (b) total level AUC_ss,central_ quantities (further details can be found in the ESI†). Both show significant linear relationships, such that those that displaying human *in vivo* efficacy at higher levels tend to have higher centroid DQI and *vice versa*. The statistics for both plots are slightly better than for the plot in Fig. 7. It can be argued that these centroid DQI values contain slightly more information than Cl for this small data set of 15 compounds. This can be understood from the perspective that oral absorption and drug distribution will have influence over *in vivo* levels in addition to that of Cl. It can be inferred from these plots that compounds with lower potency require a higher centroid DQI (total level *C*_ss,central,max_ or AUC_ss,central_). This can be understood from the perspective of a fixed dose, such that a less potent compound requires more of the oral dose to be in the central compartment at steady state to drive the therapeutic effect, whereas a more potent compound requires less of the oral dose to be in the central compartment to drive a similar effect.

Clearly, an appreciation of potency is a key factor in understanding the plots in Fig. 8. Still, the centroid DQI values for the total level *C*_ss,central,max_ or AUC_ss,central_ quantities are sufficient to explain approximately half of the information in the human *in vivo* efficacy values for the 15 compounds.

Relatedly, DQI and HLD values can be proposed as novel compound descriptors for use in conjunction with QSAR modelling methods (and other descriptors) to model other *in vivo* quantity endpoints. At the virtual design stage, such an approach would involve use of *in silico* predictions for input data for the model to derive DQI and HLD values, which could then be used as descriptors in further *in silico* models.

### Additional considerations

This study uses solubility_7.4_ data generated at room temperature, when 37 °C would be more relevant. It is reasonable to assume that solubility_7.4_ will increase with temperature in all cases, resulting in higher HLD values. Furthermore, variations in the magnitude of model parameters (*e.g.*, *V*_intestinal_, *V*_central_, *V*_terminal_ to *V*_ss_ ratio, absorption window, dosing intervals, *etc.*) will lead to variations in DQI and HLD values. However, the approach discussed here focuses on the evaluation of DQI and HLD for a set of compounds within a standardised PK model (with specific settings) and it is the relative, rather than absolute, values that matter. If absolute values are important, then correction factors are required. For example, if the *in vivo* quantity and corresponding dose for a representative set of compounds are known, DQI values could be determined using the method described in this work and eqn (6) applied to predict the doses based on the known *in vivo* quantity. Correction factors for the predicted dose can be derived from a linear regression equation between the known and predicted doses. A similar approach can be used to determine correction factors for an *in vivo* quantity.

Although this study focuses on determining DQI and HLD for the total and free level *C*_ss,central,max_ and AUC_ss,central_, other *in vivo* quantities could also be assessed using this model. These include total and free levels for the average concentration (*C*_ss,central,average_) and the minimum concentration (*C*_ss,central,min_) at steady state in compartment B. In addition, the corresponding levels in compartment C (peripheral compartment) can be considered, including steady-state total and free levels for the maximum concentration (*C*_ss,peripheral,max_), the average concentration (*C*_ss,peripheral,average_), the minimum concentration (*C*_ss,peripheral,min_) and the area under the curve AUC_ss,peripheral_.

Importantly, a twice-daily dosing scenario is considered; changing the dosing interval will lead to different DQI and HLD values for the same compound. Changing the simulation length can also affect these values, in particular if it is shortened. From a virtual drug design perspective, consideration of 14 repeat doses using a once- or twice-daily dosing scenario is recommended.

With respect to the use of 20 different dose simulations to define a dose–quantity curve, this can be reduced to two in theory. The DQI can be determined from a simulation using a very low dose (*e.g.*, 0.000001 mg), where linear PK can be assumed. The HLD can be determined from a simulation using a very high dose (*e.g.*, 10 000.0 mg), where compartment A can be assumed to be saturated throughout the absorption window and the corresponding log_10_(quantity) is at its maximum (*i.e.*, log_10_(max_quantity)). Relatedly, the standardised method used to determine the HLD only provides an approximation – other methods can be used, including fitting the dose–quantity curve to a power function of the form:

8 log_10_(quantity) = log_10_(max_quantity) – log_10_(1 + 10^HLD–log_10_(dose)^)

The application of eqn (8) using a least-fit method benefits from the use of as many data points as possible to define the transition region of the dose–quantity curve from linear to non-linear PK.

The DQI and HLD calculations can be made in different mammalian species by adjusting the PK model settings accordingly and by using species-specific *in silico* predictions for *V*_ss_, Cl and PPB (and assuming the use of *P*_app,Caco2,pH6.5_ for other species).

Finally, an extension of this study, which is beyond the scope of the present work due to its size and complexity, would be to use *in silico* QSAR models for p*K*_a_, solubility_pH7.4_, *P*_app,Caco2,pH6.5_, *V*_ss_, Cl and (optionally) PPB to predict the input data for a larger set of compounds, calculate their DQI values, and assess how well they relate to *in vivo* efficacy and toxicity data.

## Conclusions

Assessing the oral drug suitability of compounds at the virtual design stage is an important objective. This study describes a methodology that provides an alternative to the heuristic approaches that emphasise controlling a compound's physicochemical properties. The methodology attempts to simplify the evaluation of compounds based on their estimated *in vivo* quantity levels within a mammalian body. This simplification comes from the application of the compound-specific DQI and HLD values for a particular *in vivo* quantity, calculated by assessing a series of PK model simulations. In essence, the PK model takes the form a series of rate equations that can estimate the varying exposure of a compound in different parts of the body over time and repeated oral dosing; for this study, the focus was on steady-state exposure levels. An open-source Python library^31^ provides a mechanism to perform such complex calculations, also taking into consideration distribution kinetics variations and random (Gaussian) error in the values of the input data. Such functionality facilitates the use of this method at the virtual design stage, where *in silico* QSAR models can be used to provide the input data and the associated prediction errors factored into the calculation of a compound's DQI and HLD. The DQI parameter is a power term that relates an oral dose of a compound to its estimated *in vivo* quantities; for a given dose, a larger DQI value corresponds to a larger *in vivo* quantity. This is only relevant during linear PK conditions and the corresponding HLD value approximates the upper dose limit of applicability.

Application of this methodology to 15 known oral drugs demonstrates how different compounds that have vastly different tendencies in terms of absorption into, and elimination from, the body can be compared on the same scale. In spite of the computational complexities associated with gaining such insight, DQI and HLD provide a direct relationship between a compound's dose and *in vivo* exposure. Evaluation of a compound's oral drug suitability is simply dependent on the ability to match a compound's dose to the required *in vivo* exposure.

## Conflicts of interest

In carrying out this work, the author has used computational tools supplied by *InSilico*Lynx Ltd, of which he is the sole company share owner. *InSilico*Lynx Ltd is a member of the RSC Enterprise Plus program.

## Supplementary Material

Supplementary information

Supplementary information

Supplementary information

Supplementary information

Supplementary information

Supplementary information

Supplementary information

Supplementary information
