# Supplementary material for: Oral drug suitability parameters
Source: Medchemcomm. 2018 Feb 5;9(3):460–70. doi: 10.1039/c7md00586e (PMC6072407; doi:10.1039/c7md00586e)
Supplement: Supplementary file 1 [file MD-009-C7MD00586E-s001.pdf]

Electronic Supplementary Material (ESI) for MedChemComm.
This journal is © The Royal Society of Chemistry 2018

Mathematical equations for compartmental models

Below are rate equations associated to four different compartment models that can be used to approximate the quantities of a compound dosed to a mammalian body; these models being referred to in the associated article.

Intravenous 1-compartment model

Intravenous dose directly into compartment B that represents the body and from which the compound is eliminated according to the rate constant k_4.

Equation 1:

$$\frac{dx_B}{dt} = -k_4 x_B$$

Equation 2:

$$x_B = x_B^0 e^{-k_4 t}$$

where x_B^0 equals the intravenous dose (mg) and x_B the quantity (mg) of the compound in compartment B at a particular time point, t. See below for details on k_4.

Intravenous 2-compartment model

Intravenous dose directly into compartment B that represents the central compartment of the body and from which compound is eliminated according to the rate constant k_4. Compound in compartment B can also distribute into and out of compartment C that represents the peripheral compartment of the body according to the rate constants k_2 and k_3, respectively.

Equation 3:

$$\frac{dx_B}{dt} = -k_2 x_B + k_3 x_C - k_4 x_B$$

Equation 4:

$$x_B = \frac{x_B^0}{\beta - \alpha}\left[(k_3 - \alpha)e^{-\alpha t} - (k_3 - \beta)e^{-\beta t}\right]$$

Equation 5:

$$\frac{dx_C}{dt} = k_2 x_B - k_3 x_C$$

Equation 6:

$$x_C = \frac{k_2 x_B^0}{\beta - \alpha}\left[e^{-\alpha t} - e^{-\beta t}\right]$$

where x_B^0 equals the intravenous dose (mg) and x_B and x_C are the quantities (mg) of the compound in compartment B and C, respectively, at a particular time point, t. See below for details on α, β, k_2 and k_3.

Oral 1-compartment model

Extravascular dose into compartment A that represents the small intestines and from which compound is absorbed into compartment B according to the rate constant k_1. Compartment B represents the body from which compound is eliminated according to the rate constant k_4. Absorption from compartment A occurs during an absorption window during which the compartment could be:

(i) saturated throughout,
(ii) saturated at the beginning becoming unsaturated,
(iii) unsaturated throughout.

Different equations apply depending on the above absorption scenarios and time point during the simulation. Note, at the end of the absorption window only elimination occurs and different equations also apply.

Equations to use for time points when absorption can occur and compartment A is saturated

Equation 7:

$$\frac{dx_A}{dt} = -z$$

Equation 8:

$$x_A = x_A^0 - zt$$

Equation 9:

$$\frac{dx_B}{dt} = zF_h - k_4 x_B$$

Equation 10:

$$x_B = \frac{zF_h}{k_4}(1 - e^{-k_4 t})$$

where x_A^0 equals the oral dose (mg) and x_A and x_B are the quantities (mg) of the compound in compartment A and B, respectively, at a particular time point, t. See below for details on z, F_h and k_4.

Equations to use for time points when absorption can occur and compartment A is unsaturated

Equation 11:

$$\frac{dx_A}{dt} = -k_1 x_A$$

Equation 12:

$$x_A = x_A^0 e^{-k_1 t}$$

Equation 13:

$$\frac{dx_B}{dt} = F_h k_1 x_A - k_4 x_B$$

Equation 14:

$$x_B = \frac{F_h k_1 x_A^0}{k_4 - k_1}(e^{-k_1 t} - e^{-k_4 t}) + x_B^0 e^{-k_4 t}$$

where x_A^0 equals the oral dose (mg) and x_A and x_B are the quantities (mg) of the compound in compartment A and B, respectively, at a particular time point, t. See below for details on F_h, k_1.and k_4. If throughout the absorption window compartment A was unsaturated then x_B^0 is zero otherwise it is given by Equation 10 at the time point corresponding to the transition from a saturated to unsaturated state with respect to compartment A.

Equations to use for time points when absorption has ceased

Equation 15:

$$\frac{dx_B}{dt} = -k_4 x_B$$

Equation 16:

$$x_B = x_B^0 e^{-k_4 t}$$

where x_B is the quantity (mg) of the compound in compartment B at a particular time point, t. Depending on the saturation state of compartment A at the end of the absorption window, x_B^0 is given by Equation 10 or 14 at the time point corresponding to end of the absorption window. See below for details on k_4.

Oral 2-compartment

Extravascular dose into compartment A that represents the small intestines and from which compound is absorbed into compartment B according to the rate constant k_1. Compartment B represents the body from which compound is eliminated according to the rate constant k_4. Compound in compartment B can also distribute into and out of compartment C that

represents the peripheral compartment of the body according to the rate constants k_2 and k_3, respectively. Absorption from compartment A occurs during an absorption window during which the compartment could be:

(i) saturated throughout,
(ii) saturated at the beginning becoming unsaturated,
(iii) unsaturated throughout.

Different equations apply depending on the above absorption scenarios and time point during the simulation. Note, at the end of the absorption window only elimination occurs and different equations also apply.

Equations to use for time points when absorption can occur and compartment A is saturated

Equation 17:

$$\frac{dx_A}{dt} = -z$$

Equation 18:

$$x_A = x_A^0 - zt$$

Equation 19:

$$\frac{dx_B}{dt} = zF_h - k_2 x_B + k_3 x_C - k_4 x_B$$

Equation 20:

$$x_B = zF_h \left[\frac{k_3}{\alpha\beta} - \frac{(k_3 - \alpha)e^{-\alpha t}}{\alpha(\beta - \alpha)} + \frac{(k_3 - \beta)e^{-\beta t}}{\beta(\beta - \alpha)} \right]$$

Equation 21:

$$\frac{dx_C}{dt} = k_2 x_B - k_3 x_C$$

Equation 22:

$$x_C = zF_h k_2 \left[\frac{1}{\alpha\beta} - \frac{e^{-\alpha t}}{\alpha(\beta - \alpha)} + \frac{e^{-\beta t}}{\beta(\beta - \alpha)} \right]$$

where x_A^0 equals the oral dose (mg) and x_A, x_B and x_C are the quantities (mg) of the compound in compartment A, B and C, respectively, at a particular time point, t. See below for details on z, F_h, α, β, k_2 and k_3.

Equations to use for time points when absorption can occur and compartment A is unsaturated

Equation 23:

$$\frac{dx_A}{dt} = -k_1 x_A$$

Equation 24:

$$x_A = x_A^0 e^{-k_1 t}$$

Equation 25:

$$\frac{dx_B}{dt} = F_h k_1 x_A - k_2 x_B + k_3 x_C - k_4 x_B$$

Equation 26:

$$x_B = F_h k_1 x_A^0 \left[\frac{(k_3 - k_1)e^{-k_1 t}}{(\alpha - k_1)(\beta - k_1)} - \frac{(k_3 - \alpha)e^{-\alpha t}}{(\alpha - k_1)(\beta - \alpha)} + \frac{(k_3 - \beta)e^{-\beta t}}{(\beta - k_1)(\beta - \alpha)} \right] + \frac{x_B^0}{\beta - \alpha}\left[(k_3 - \alpha)e^{-\alpha t} - (k_3 - \beta)e^{-\beta t}\right] + \frac{k_3 x_C^0}{\beta - \alpha}\left(e^{-\alpha t} - e^{-\beta t}\right)$$

Equation 27:

$$\frac{dx_C}{dt} = k_2 x_B - k_3 x_C$$

Equation 28:

$$x_C = \frac{k_2 x_B^0}{\beta - \alpha}\left(e^{-\alpha t} - e^{-\beta t}\right) + x_C^0 e^{-k_3 t} - k_2 k_3 x_C^0 \left[\frac{(\beta - \alpha)e^{-k_3 t} + (k_3 - \beta)e^{-\alpha t} + (\alpha - k_3)e^{-\beta t}}{(\alpha - k_3)(\beta - \alpha)(k_3 - \beta)} \right] - F_h k_1 k_2 x_A^0 \left[\frac{(\beta - \alpha)e^{-k_1 t} + (k_1 - \beta)e^{-\alpha t} + (\alpha - k_1)e^{-\beta t}}{(\alpha - k_1)(\beta - \alpha)(k_1 - \beta)} \right]$$

where x_A^0 equals the oral dose (mg) and x_A, x_B and x_C are the quantities (mg) of the compound in compartment A, B and C, respectively, at a particular time point, t. See below for details on F_h, α, β, k_1, k_2 and k_3. If throughout the absorption window compartment A was unsaturated then x_B^0 and x_C^0 are zero otherwise they are given by Equations 20 and 22, respectively, at the time point corresponding to the transition from a saturated to unsaturated state with respect to compartment A.

Equations to use for time points when absorption has ceased

Equation 29:

$$\frac{dx_B}{dt} = -k_2 x_B + k_3 x_C - k_4 x_B$$

Equation 30:

$$x_B = \frac{k_3 x_C^0}{\beta - \alpha}\left(e^{-\alpha t} - e^{-\beta t}\right) + \frac{x_B^0}{\beta - \alpha}\left[(k_3 - \alpha)e^{-\alpha t} - (k_3 - \beta)e^{-\beta t}\right]$$

Equation 31:

$$\frac{dx_C}{dt} = k_2 x_B - k_3 x_C$$

Equation 32:

$$x_C = x_C^0 e^{-k_3 t} + \frac{k_2 x_B^0}{\beta - \alpha}\left(e^{-\alpha t} - e^{-\beta t}\right) - k_2 k_3 x_C^0 \left[\frac{(\beta - \alpha)e^{-k_3 t} + (k_3 - \beta)e^{-\alpha t} + (\alpha - k_3)e^{-\beta t}}{(\alpha - k_3)(\beta - \alpha)(k_3 - \beta)}\right]$$

where x_B and x_C are the quantities (mg) of the compound in compartment B and C, respectively, at a particular time point, t. See below for details on α, β, k_2 and k_3. Depending on the saturation state of compartment A at the end of the absorption window, x_B^0 is given by Equation 20 or 26 at the time point corresponding to end of the absorption window. Similarly, depending on the saturation state of compartment A at the end of the absorption window, x_C^0 is given by Equation 22 or 28 at the time point corresponding to end of the absorption window.

Additional terms

Saturated absorption rate (z):

Equation 33:

$$z = k_1 \cdot solubility_{pH6.5} \cdot mwt \cdot V_{intestinal}$$

where $solubility_{pH6.5}$ (mol L^{-1}) is a compound's aqueous solubility at pH 6.5, mwt is the compound's molecular weight and $V_{intestinal}$ is the intestinal fluid volume (80 mL).

Fraction escaping first pass metabolism (F_h):

Equation 34:

$$F_h = 1 - E_h = 1 - \frac{Cl}{Q_h}$$

where E_h is the compound's hepatic extraction ratio, Cl is the compound's *in vivo* clearance (L min^{-1}) and Q_h is the liver blood flow (L min^{-1}).

The α and β terms:

The following two equations relate the α and β terms to k_2, k_3 and k_4:

Equation 35:

$$\alpha + \beta = k_2 + k_3 + k_4$$

Equation 36:

$$\alpha\beta = k_3 k_4$$

Rate constant: k_1 (min^{-1})

See the associated article for details on calculating k_1.

Rate constant: k_4 (min^{-1})

From the perspective of a 1-compartment model for the body, k_4 can be defined as:

Equation 37:

$$k_4 = \frac{Cl}{V_{ss}}$$

where V_{ss} is the compound's steady state volume of distribution (L).

From the perspective of a 2-compartment model for the body (*i.e.*, central and peripheral compartment), k_4 can be defined as:

Equation 38:

$$k_4 = \frac{Cl}{V_{central}}$$

where $V_{central}$ is the compound's initial dilution volume (L).

Rate constants: k_2 (min^{-1}) and k_3 (min^{-1})

From the perspective of a 2-compartment model for the body (*i.e.*, central and peripheral compartment), Cl can be defined as:

Equation 39:

$$Cl = \beta V_{terminal}$$

where $V_{terminal}$ is the compound's terminal volume of distribution (L).

Defining p as the ratio of $V_{terminal}$ to V_{ss}:

Equation 40:

$$\frac{V_{terminal}}{V_{ss}} = p$$

where $p >= 1$.

Substituting p into Equation 39 and rearranging gives:

Equation 41:

$$\beta = \frac{Cl}{pV_{ss}}$$

Furthermore, V_{ss} can be defined as:

Equation 42:

$$V_{ss} = V_{central}\left(1 + \frac{k_2}{k_3}\right)$$

Equation 42 can be rearranged to give:

Equation 43:

$$\frac{k_2}{k_3} = \frac{V_{ss}}{V_{central}} - 1 = q$$

Equations 35 and 36 can be combine to form quadratic equations whose solutions are given by:

Equation 44:

$$\alpha = \frac{1}{2}\left[(k_2 + k_3 + k_4) + \sqrt{(k_2 + k_3 + k_4)^2 - 4k_3k_4}\right]$$

Equation 45:

$$\beta = \frac{1}{2}\left[(k_2 + k_3 + k_4) - \sqrt{(k_2 + k_3 + k_4)^2 - 4k_3k_4}\right]$$

Rearranging Equation 43 and substituting into Equation 45 and subsequently rearranging gives:

Equation 46:

$$k_3 = \frac{\left(\beta - \frac{\beta^2}{k_4}\right)}{\left[1 - \frac{\beta(1+q)}{k_4}\right]}$$

Based upon Equation 43, Equation 46 can be rearranged to:

Equation 47:

$$k_3 = \frac{\left(\beta - \frac{\beta^2}{k_4}\right)}{\left[1 - \frac{\beta\left(\frac{V_{ss}}{V_{central}}\right)}{k_4}\right]}$$

Equations 38, 41 and 47 can be combined along with a compound's Cl, $V_{central}$, V_{ss} and $V_{terminal}$ to calculate k_3. Subsequently, k_2 can be determined by rearranging and solving Equation 43.
